# *Klebsiella pneumoniae* Infections in Dogs: A One Health Review of Antimicrobial Resistance, Virulence Factors, Zoonotic Risk, and Emerging Alternatives

**DOI:** 10.3390/microorganisms14010149

**Published:** 2026-01-09

**Authors:** Mălina Lorena Mihu, George Cosmin Nadăş, Cosmina Maria Bouari, Nicodim Iosif Fiț, Sorin Răpuntean

**Affiliations:** Department of Microbiology, Immunology and Epidemiology, Faculty of Veterinary Medicine, University of Agricultural Sciences and Veterinary Medicine, 400372 Cluj-Napoca, Romania; malina.mihu@usamvcluj.ro (M.L.M.); cosmina.bouari@usamvcluj.ro (C.M.B.); nfit@usamvcluj.ro (N.I.F.); sorin.rapuntean@usamvcluj.ro (S.R.)

**Keywords:** *Klebsiella pneumoniae*, dogs, antimicrobial resistance, zoonosis, virulence factors, One Health, companion animals, hypervirulent strains

## Abstract

*Klebsiella pneumoniae* is increasingly reported in canine medicine, with growing attention to multidrug-resistant (MDR) and hypervirulent strains. Although its overall prevalence in dogs appears relatively low, published studies indicate that affected animals may harbor clinically important resistance determinants, including extended-spectrum β-lactamases and, less frequently, carbapenemases. Canine isolates described in the literature also carry virulence-associated traits such as hypermucoviscosity and enhanced iron-acquisition systems, which overlap with features of high-risk human lineages and suggest potential, but largely inferred, interspecies links. These observations highlight the relevance of a One Health perspective and the importance of coordinated surveillance that includes companion animals. This narrative review synthesizes available literature on the epidemiology, clinical presentations, antimicrobial resistance, virulence traits, and molecular characteristics of *K. pneumoniae* in dogs. We critically evaluate evidence suggesting that dogs may function as reservoirs, sentinels, or amplifiers of MDR strains, particularly in clinical settings or following antimicrobial exposure. In addition, we summarize emerging alternative and adjunctive strategies—such as bacteriophage therapy, antimicrobial peptides, anti-virulence approaches, microbiome-based interventions, as well as strengthened antimicrobial stewardship and infection-control practices—that are under investigation as complements to conventional antibiotics. Overall, published evidence indicates that *K. pneumoniae* infections in dogs represent an under recognized but potentially important clinical and One Health concern. Continued surveillance, responsible antimicrobial use, and rigorous evaluation of non-antibiotic strategies will be essential to inform future veterinary practice and public health policy.

## 1. Introduction

*Klebsiella pneumoniae* is a Gram-negative, encapsulated bacterium recognized as a significant opportunistic pathogen in humans, causing a range of infections including pneumonia, urinary tract infections, and septicemia. Its clinical importance has intensified due to the global rise in multidrug-resistant (MDR) and hypervirulent strains [1,2]. Notably, *K. pneumoniae* is classified among the ESKAPE pathogens—*Enterococcus faecium*, *Staphylococcus aureus*, *Klebsiella pneumoniae*, *Acinetobacter baumannii*, *Pseudomonas aeruginosa*, and *Enterobacter* spp.—a group of bacteria responsible for the majority of healthcare-associated infections and characterized by a pronounced capacity to evade the effects of antimicrobial agents [3]. In veterinary medicine, particularly concerning companion animals like dogs, *K. pneumoniae* has been less extensively studied. However, recent findings indicate that dogs can harbor and potentially transmit virulent or resistant strains, raising concerns about zoonotic transmission [4,5].

In dogs, *K. pneumoniae* has been implicated in various clinical infections, including respiratory, urinary, and wound infections [4,5]. These infections can be challenging to treat due to increasing antimicrobial resistance [6,7]. The close relationship between humans and companion animals presents a unique One Health challenge. Studies have reported genetic similarities between *K. pneumoniae* strains isolated from humans and those from dogs, suggesting possible interspecies transmission [4,6,8].

Despite increasing reports of *K. pneumoniae* infections in dogs, there is a lack of consolidated literature assessing its prevalence, resistance patterns, virulence mechanisms, and zoonotic risk. Understanding these aspects is crucial for both veterinary care and public health preparedness. As a consequence, *K. pneumoniae* represents a major public health threat and a critical priority for antimicrobial resistance surveillance and control efforts worldwide [9,10].

Within the broader ecology of *K. pneumoniae*, dogs should not be viewed solely as passive extensions of the human antimicrobial resistance problem. Available evidence suggests that dogs may occupy multiple ecological roles depending on context. Studies demonstrating shared sequence types, resistance determinants, and plasmid profiles between canine and human isolates support the interpretation of dogs as spillover hosts following exposure to human-associated strains, particularly in household or clinical environments [4,8]. At the same time, colonization studies indicate that dogs may function as sentinel species, reflecting local circulation of multidrug-resistant lineages without necessarily developing disease [8,9]. Under specific conditions, such as hospitalization, antimicrobial exposure, or invasive infection, dogs may also act as reservoirs or amplifiers of clinically relevant MDR or hypervirulent *K. pneumoniae* strains, as suggested by genomic and clinical reports from veterinary settings [5,6,7]. Framing canine *K. pneumoniae* infections within this dynamic One Health context helps integrate antimicrobial resistance, virulence, and zoonotic considerations while highlighting the need for further studies to clarify transmission pathways and host-specific clinical relevance.

This review aims to (i) summarize current knowledge on the epidemiology, clinical manifestations, and antimicrobial resistance of *K. pneumoniae* in dogs, (ii) highlight the genetic and virulence characteristics of canine isolates, (iii) discuss the zoonotic potential of *K. pneumoniae* within the One Health framework and (iv) identify gaps in current research and suggest future directions.

## 2. Literature Search Strategy and Study Selection

This narrative review was conducted following established principles for scoping and narrative synthesis. A comprehensive literature search was performed across PubMed/MEDLINE, Embase, Web of Science Core Collection, Scopus, and CAB Abstracts, supplemented by manual searches of reference lists from relevant articles and reviews. Searches covered the period from 1 January 2000 to 23 September 2025, reflecting two decades of emerging data on *Klebsiella pneumoniae* infections in companion animals, especially dogs. No restrictions on country or study design were applied during initial retrieval.

Search strategies were tailored to each database using controlled vocabulary (e.g., MeSH in PubMed; Emtree in Embase) combined with free-text keywords. The core search string included combinations of “*Klebsiella pneumoniae*”, “dog”, “dogs”, “canine”, “canines”, “infection”, “clinical isolates”, “antimicrobial resistance”, “AMR”, “ESBL”, “carbapenemase”, “virulence”, “hypervirulent”, “hvKp”, “zoonotic”, “One Health”, “companion animals”, or “small animals”.

Studies were included if they met the following criteria: Language: English. Species: Domestic dogs (*Canis lupus familiaris*). Studies including multiple species were eligible if canine data were extractable. Study type: Clinical studies, surveillance reports, case reports/series, molecular epidemiology studies, experimental infection studies, genomic analyses, or reviews providing primary data relevant to canine *K. pneumoniae*. Content: Must report at least one of the following: isolation or detection of *K. pneumoniae* in dogs, antimicrobial susceptibility or resistance profiles, virulence factors or hypervirulent phenotypes, molecular typing (e.g., MLST, WGS), zoonotic transmission or One Health aspects.

Exclusion criteria were: non-canine studies where dog-specific results could not be extracted, reviews without primary data (unless they provided significant contextual references), studies focused exclusively on *Klebsiella* species other than *K. pneumoniae*, non-clinical experimental microbiology without relevance to animal infections.

All search results were imported into a reference manager. Duplicate records were identified and removed using automated detection, followed by manual verification. Titles and abstracts were screened for eligibility, with full texts retrieved when relevance was uncertain. Reference lists of all eligible studies and recent reviews were manually examined to identify additional publications not captured by database searches.

## 3. Clinical Spectrum and Epidemiology in Dogs

The epidemiology of *K. pneumoniae* in dogs is still not fully understood due to limited large-scale surveillance studies. Most available data come from isolated case reports or small cohort studies, often in veterinary clinical settings. Reports from various countries, including China, Germany, and Italy, indicate that *K. pneumoniae* is increasingly encountered in veterinary microbiology laboratories, primarily from dogs with infections of the urinary tract, respiratory system, and skin wounds [4,7,11].

In clinical cases, *K. pneumoniae* is often isolated as part of polymicrobial infections, making it difficult to establish its role as a primary pathogen. Some studies have reported isolation rates ranging from 0.4% to 1.2% among bacterial cultures obtained from dogs with clinical signs of infection [11]. Additionally, studies have explored *K. pneumoniae* colonization in healthy dogs, indicating that dogs may serve as silent carriers [8]. *K. pneumoniae* has been isolated from a variety of clinical and colonization-related samples in dogs, including urine, respiratory tract specimens, wound exudates, feces, and, less frequently, blood, depending on the clinical presentation and study design [10].

Recent studies have further highlighted the variability in *K. pneumoniae* prevalence depending on geographic region and clinical context. For example, Ribeiro et al. [12] reported that *K. pneumoniae* represented 6.7% of all bacterial isolates obtained from canine infections over a 20-year period in Brazil, particularly in cases of otitis and wound infections. Similarly, Hyeon et al. [7] identified the bacterium in 1.7% of clinical samples from dogs in South Korea, often exhibiting multidrug resistance and co-resistance to third-generation cephalosporins and fluoroquinolones. In healthy populations, Carvalho et al. [13] detected ESBL-producing *K. pneumoniae* in rectal swabs from asymptomatic dogs in urban settings, including strains carrying high-risk clones such as ST11 and ST15. These findings support the notion that healthy dogs may serve as reservoirs of clinically significant *K. pneumoniae* strains, contributing to potential zoonotic transmission and antimicrobial resistance dissemination.

Several factors have been associated with increased risk of *K. pneumoniae* infection or colonization in dogs, including prior antimicrobial therapy, hospitalization or recent surgery, presence of indwelling medical devices, immunosuppression, and close contact with humans, especially in multi-pet households or urban environments [4,7].

Recent years have seen a growing number of reports of multidrug-resistant and hypervirulent *K. pneumoniae* strains in canine isolates from (1) China: reports of carbapenem-resistant *K. pneumoniae* with high virulence genes [8], (2) Germany: comprehensive data showing resistance trends in companion animals over a three-year period [11], and (3) Italy: clonal transmission of MDR strains between dogs and humans in households [4].

Further surveillance studies have expanded our understanding of *K. pneumoniae* distribution in canine populations across other regions. In South Korea, Hyeon et al. [7] reported the detection of MDR *K. pneumoniae* strains with resistance to multiple antibiotic classes, including extended-spectrum β-lactams and aminoglycosides. In Brazil, Sellera et al. [14] documented the emergence of a hypermucoviscous CG258 strain harboring the *bla*_KPC-2 gene in a domestic dog, signaling the presence of globally relevant high-risk clones in South America. Meanwhile, in the Caribbean, a One Health genomic study by Butaye et al. [15] revealed overlapping strain types in animals and humans, highlighting zoonotic exchange risks in smaller, interconnected communities. These reports collectively emphasize a global pattern of increasingly diverse and resistant *K. pneumoniae* populations in dogs, often paralleling trends observed in human healthcare settings. These findings highlight the importance of including companion animals in antimicrobial resistance monitoring programs as part of a One Health surveillance strategy.

It is important to distinguish between colonization and clinical infection when interpreting reports of *K. pneumoniae* in dogs [8]. In many studies, isolates are recovered from fecal samples, skin, or mucosal surfaces without clear evidence of disease, indicating asymptomatic carriage rather than infection [12]. By contrast, true clinical infections, such as urinary tract infections, pneumonia, wound infections, or septicemia, are reported less frequently and are often based on sporadic case reports or small case series [6]. Consequently, the clinical significance of *K. pneumoniae* in dogs should be interpreted cautiously, particularly when prevalence data are derived from colonization studies rather than confirmed disease [8].

Similarly, while hypervirulence-associated traits and genes have been identified in some canine isolates, their clinical relevance in dogs remains unclear. Most evidence linking hypervirulence to severe disease outcomes is derived from human or experimental studies, and direct associations between hypervirulent genotypes and increased pathogenicity in dogs are limited [9,10,12]. Further studies are needed to clarify whether hypervirulence markers confer the same clinical impact in canine hosts as observed in humans.

## 4. Pathogenesis and Clinical Manifestations in Canines

*K. pneumoniae* infections in dogs can present in a variety of anatomical sites. While often considered an opportunistic pathogen, under certain conditions, it can act as a primary agent of disease, especially in immunocompromised or hospitalized dogs. The most commonly reported clinical syndromes include (1) urinary tract infections (UTIs), particularly associated with female dogs, recurrent UTIs, or those with indwelling urinary catheters [7]; (2) respiratory tract infections: infections range from upper respiratory colonization to bronchopneumonia, often seen in shelter dogs or those in intensive care units [6]; (3) wound and skin infections: post-surgical wounds or traumatic injuries can become infected, especially in the presence of MDR strains [11]; (4) septicemia and systemic infections: though rare, systemic infections have been documented, particularly in puppies, elderly dogs, or those undergoing immunosuppressive therapy [6]; (5) otitis and pyometra: occasionally isolated from ear infections or uterine infections, either alone or as part of a polymicrobial flora [11].

Clinical signs of *K. pneumoniae* infection in dogs are highly variable and depend on the anatomical site affected, the virulence of the strain, and the host’s immune status. Commonly reported clinical presentations include the following:

1. Urinary tract infections (UTIs): Dysuria, hematuria, pollakiuria, and malodorous urine. Some cases progress to pyelonephritis, with systemic signs like fever and vomiting [7].
2. Respiratory infections: Coughing, nasal discharge, dyspnea, lethargy, and abnormal lung sounds. Severe cases may present with bronchopneumonia and radiographic changes [6].
3. Skin and wound infections: Local swelling, purulent discharge, inflammation, delayed wound healing, or necrosis. Post-surgical infections can present similarly [11].
4. Septicemia and systemic illness: Fever, tachycardia, hypotension, shock, and multi-organ dysfunction in acute systemic infections. These are often associated with hypervirulent strains [5].
5. Ear infections and pyometra: Pain, discharge, head shaking (otitis externa), or purulent vaginal discharge and abdominal pain in reproductive tract infections [16,17,18].

Because many of these signs are nonspecific and overlap with infections caused by other bacteria, microbiological confirmation is essential for accurate diagnosis and appropriate treatment [7,19]. Although *K. pneumoniae* infections in dogs are often opportunistic, severe disease has been reported. Rare fatal outcomes have been described in cases of septicemia and severe enteritis, particularly in puppies or immunocompromised animals [11]. In contrast, several studies report serious infections, including urinary tract infections, pneumonia, and bacteremia, that were successfully treated and resolved following appropriate antimicrobial therapy guided by susceptibility testing [5,6].

The ability of *K. pneumoniae* to establish infection and cause disease in dogs depends on a complex array of virulence factors. These mechanisms are shared with human strains and are increasingly being identified in isolates from veterinary sources [20,21,22,23,24].

One of the most critical virulence factors, the capsule is composed of polysaccharides that help evade host immune responses by preventing phagocytosis and complement activation. Certain capsular serotypes, especially K1 and K2, are frequently associated with hypervirulent strains and have been identified in some canine isolates [25].

Hypermucoviscosity phenotype (HMV) is conferred by genes like *rmp*A and *rmp*A2, leading to increased capsule production and a sticky, viscous appearance on culture media. It is strongly linked with enhanced virulence, bacteremia, and metastatic infections [5,7].

Fimbriae (type 1 and type 3) facilitate adhesion to epithelial surfaces, particularly in the urinary and respiratory tracts. Type 3 fimbriae also support biofilm formation on abiotic surfaces such as catheters [26], increasing persistence and resistance.

*K. pneumoniae* produces multiple siderophores (e.g., enterobactin, aerobactin, salmochelin, yersiniabactin) to scavenge iron from host tissues, which is crucial for survival in iron-limited environments like the bloodstream [27]. Aerobactin production, in particular, is associated with hypervirulent strains. Lipopolysaccharide (LPS) is a component of the outer membrane that can stimulate intense inflammatory responses and contribute to septic shock in systemic infections [2].

Many MDR strains harbor plasmids that encode both resistance and virulence genes (e.g., *bla*_KPC, *bla*_CTX-M, *rmp*A), allowing them to evade both host defenses and antimicrobial therapy [4,8]. Co-localization of these genes may explain the increasing severity of infections in veterinary settings. The expression of these factors can vary among strains and may correlate with clinical severity. Some canine isolates have shown genotypic and phenotypic characteristics similar to those of human hypervirulent strains, raising concerns about zoonotic potential [4,22,24,28,29].

Recent whole-genome sequencing (WGS) studies have shown that *K. pneumoniae* isolates from dogs can share sequence types (STs) with human clinical strains, such as ST11, ST15, and ST307 [5,8]. These STs are often associated with multidrug resistance and global outbreaks in human medicine, supporting concerns over zoonotic potential and cross-species transmission. Moreover, plasmid analysis has revealed the presence of conjugative plasmids capable of transferring both resistance and virulence determinants between bacterial strains and across species [25]. This reinforces the role of companion animals as potential reservoirs or bridging hosts in the transmission of high-risk clones.

## 5. Antimicrobial Resistance Patterns of *Klebsiella pneumoniae* in Dogs

*K. pneumoniae* is one of the most concerning bacterial species in the context of antimicrobial resistance (AMR), both in human and veterinary medicine. Its capacity to acquire and disseminate resistance genes, often via mobile genetic elements like plasmids and integrons, makes it a formidable pathogen. In dogs, resistant *K. pneumoniae* strains have been increasingly reported from clinical infections, particularly in hospital settings and among patients with prior antibiotic exposure [4,11].

The most commonly identified resistance mechanisms in canine isolates include (i) extended-spectrum β-lactamases (ESBLs): e.g., *bla*_CTX-M, *bla*_SHV, and *bla*_TEM [13,30,31,32], (ii) carbapenemases: *bla*_KPC, *bla*_NDM, and *bla*_OXA-48—rare but of major concern when present [5,33,34], (iii) aminoglycoside-modifying enzymes: e.g., *aac*(6′)-Ib, and (iv) efflux pumps and porin mutations: Contributing to multidrug resistance beyond β-lactams [35,36,37].

Recent studies have highlighted the growing prevalence of MDR *K. pneumoniae* in dogs. MDR is commonly defined as resistance to at least one agent in three or more antimicrobial classes. Frenzer et al. [11] reported that over 50% of *K. pneumoniae* isolates from dogs in Germany exhibited resistance to third-generation cephalosporins, fluoroquinolones, and aminoglycosides. Pachanon et al. [5] identified ESBL-producing *K. pneumoniae* in both clinical and fecal isolates from dogs in China, with some strains also showing carbapenem resistance, an alarming finding considering the drug’s use as a last resort. Liu et al. [9] detected co-carriage of *bla*_KPC-2, *bla*_NDM-1, and *rmp*A genes in a single canine isolate, suggesting convergence of resistance and virulence traits.

In most cases, isolates displayed resistance to β-lactams, including penicillins and cephalosporins, fluoroquinolones, such as enrofloxacin and ciprofloxacin, aminoglycosides, including gentamicin and amikacin, as well as tetracyclines and sulfonamides. Though not first-line agents, resistance to these drugs further limits treatment options [11,30,38,39,40,41,42,43,44].

The detection of resistance genes in *K. pneumoniae* from dogs that are genetically similar or identical to those found in human clinical isolates raises concerns about zoonotic AMR transmission. Shared sequence types (e.g., ST11, ST15, ST147) and plasmid profiles between dog and human isolates have been documented in several studies [4,5,8]. Horizontal gene transfer through conjugative plasmids is a key mechanism by which resistance can spread between bacteria in different hosts. Direct transmission between dogs and humans is plausible, especially in households where close contact is frequent, or in veterinary hospitals where infection control measures may be insufficient.

The presence of MDR *K. pneumoniae* in dogs significantly complicates therapeutic management. Empirical treatment may fail unless guided by culture and susceptibility results [45,46,47]. Moreover, many first-line veterinary antibiotics are becoming ineffective against resistant strains [48,49]. Carbapenems, though rarely used in veterinary medicine due to stewardship concerns, are sometimes the only effective drugs in vitro [50,51,52]. However, their use should be extremely restricted [53].

Combination therapies (e.g., β-lactam with clavulanic acid) may offer some efficacy against ESBL-producers but are ineffective against carbapenemase-producers [49,54]. Therapeutic failure, prolonged infections, and increased risk of dissemination to other animals or humans are major concerns [53,55]. These clinical challenges underline the importance of antimicrobial stewardship in veterinary practice [48,53].

A coordinated approach to surveillance is critical for monitoring AMR trends and guiding appropriate use of antibiotics. However, such programs are still lacking or underdeveloped in the veterinary field, particularly for companion animals [4,6,8,20,56,57,58].

Recommendations include routine antimicrobial susceptibility testing (AST) in all suspected bacterial infections, limiting the use of critically important antimicrobials (CIAs) such as carbapenems and third-generation cephalosporins, implementing infection control practices in veterinary hospitals to prevent nosocomial transmission, and inclusion of pets in One Health AMR surveillance networks to capture the true scope of resistance dissemination [59,60].

## 6. Virulence Factors and Genetic Characterization

*K. pneumoniae* possesses a broad arsenal of virulence factors that contribute to its ability to colonize, evade host defenses, and cause invasive infections. In canine isolates, many of these factors mirror those observed in human strains, reinforcing the potential for cross-species transmission and the importance of a One Health perspective [9,10,23,61,62].

Among the most significant virulence attributes are as follows: (i) Capsular polysaccharide (K antigen): The capsule is crucial for resistance to phagocytosis and serum killing. Serotypes K1 and K2 are frequently associated with hypervirulence [25]. (ii) Hypermucoviscosity phenotype: Encoded by *rmp*A and *rmp*A2, this trait enhances immune evasion and is often observed in highly invasive strains isolated from both humans and dogs [5,13]. (iii) Siderophore production: Virulent strains often carry genes encoding multiple iron-acquisition systems, including aerobactin, enterobactin, salmochelin, and yersiniabactin, which are associated with increased fitness in iron-limited environments [27]. (iv) Fimbriae (types 1 and 3): These surface structures promote adhesion to host tissues and support biofilm formation, especially relevant in urinary and respiratory infections [26].

Genomic analyses of canine isolates have revealed the presence of international high-risk clones such as ST11, ST15, and ST147, commonly implicated in human nosocomial outbreaks [4,8]. WGS has also confirmed the co-localization of resistance and virulence genes on plasmids, suggesting that dogs can serve as reservoirs for strains with enhanced pathogenic potential [5]. The detection of shared sequence types and plasmid profiles between human and animal isolates highlights the importance of continued molecular surveillance across species boundaries.

Table 1 summarizes the major antimicrobial resistance determinants and virulence factors identified in *K. pneumoniae*, highlighting their associated genes, clinical relevance, and key supporting references. Key components include the capsular polysaccharide (K antigen), which protects against phagocytosis (e.g., *wzi*, *mag*A); the hypermucoviscosity phenotype regulated by *rmp*A and *rmp*A2; fimbriae (*fim*, *mrk*) that promote adhesion and biofilm formation; and siderophores (*iuc*, *ent*, *iro*, *ybt*) for iron acquisition [9,10,23,61]. These virulence traits contribute to immune evasion, tissue colonization, and systemic spread in canine hosts and are shared with hypervirulent strains isolated from humans. Capsular polysaccharide (K antigen): Encoded by genes such as *wzi* and *mag*A, the capsule forms a protective outer layer that enhances resistance to phagocytosis and complement-mediated killing. Capsular types K1 and K2 are strongly associated with hypervirulent strains [9,10,29].

## 7. Zoonotic Potential and One Health Implications

*K. pneumoniae* poses a growing concern as a zoonotic and reverse-zoonotic pathogen, particularly due to its increasing detection in human and animal hosts. The close interaction between people and companion animals such as dogs provides an ecological niche for microbial exchange, with implications for antimicrobial resistance (AMR), virulence, and public health under the One Health umbrella [49,62,63,64,65].

Although numerous studies have demonstrated close genetic relatedness between *K. pneumoniae* isolates from dogs and humans, such as shared sequence types, resistance genes, and plasmid backbones, direct evidence of zoonotic or reverse-zoonotic transmission remains limited. In most cases, transmission is inferred based on molecular overlap rather than proven by epidemiological linkage or longitudinal transmission studies. Consequently, the zoonotic role of dogs should be interpreted as potential or presumed, pending further studies specifically designed to demonstrate direct transmission routes [4,5,6,8].

Several recent studies highlight significant genetic and phenotypic overlap between *Klebsiella pneumoniae* isolates from dogs and those causing human infections, raising concerns about bidirectional transmission and shared high-risk clones:

• Marques et al. [4] reported *K. pneumoniae* ST15 and ST147 in dogs and their owners, exhibiting nearly identical ESBL profiles, consistent with household-level transmission.
• Chen et al. [8] identified shared IncFII and IncR plasmid types in human–canine paired isolates, carrying *bla*_CTX-M-15, *qnr*B, and *sul*1, suggesting interspecies dissemination of multidrug-resistant plasmids.
• Pachanon et al. [5] documented carbapenem- and colistin-resistant *K. pneumoniae* in dogs, harboring *bla*_NDM-1, *bla*_OXA-181, and *mcr*-like resistance determinants, with genomic clustering closely related to human clinical strains in Southeast Asia.
• Liu et al. [9] described a canine hypervirulent, carbapenem-resistant ST11 strain genetically similar to human nosocomial outbreak lineages, carrying *rmp*A2, *iuc*A, and *bla*_KPC-2.
• Frenzer et al. [11] observed increasing MDR *K. pneumoniae* prevalence in European veterinary clinics, with genotypes matching those from human hospitals in the same region.

Multiple risk factors contribute to the transmission of *K. pneumoniae* between humans and dogs: (i) close cohabitation and physical contact, such as petting, licking, and shared sleeping spaces [4,8], (ii) prior antimicrobial use in pets, especially broad-spectrum antibiotics, is a well-known driver of resistance development [11], (iii) veterinary clinical environments that lack robust infection control can serve as hotspots for MDR transmission [60], and (iv) immunocompromised individuals, including the elderly or those with chronic conditions, may be particularly susceptible to opportunistic infections [58]. These risks highlight the need for comprehensive management strategies at the human–animal-environment interface.

The One Health concept emphasizes the interconnectedness of human, animal, and environmental health. In the context of *Klebsiella pneumoniae*, this framework is particularly relevant, as increasing molecular evidence reveals overlapping genotypes, including sequence types, plasmid profiles, and resistance/virulence genes, between canine and human isolates [4,6,8,20,22,56,57]. The emergence of multidrug-resistant (MDR) and hypervirulent *K. pneumoniae* strains in companion animals suggests these hosts may act not only as reservoirs but also as sentinels and amplifiers of clinically significant pathogens [49,62,63,64,65].

The bidirectional transmission of *K. pneumoniae*, from humans to pets and vice versa, has been supported by comparative genomic analyses, which have identified shared high-risk clones (e.g., ST11, ST15, ST147) and resistance determinants such as *bla*_CTX-M, *bla*_KPC, and *rmp*A [8,9]. Household settings, veterinary clinics, and animal shelters provide close-contact environments that facilitate this exchange, posing a direct challenge to infection control and antimicrobial stewardship in both sectors [66].

Currently, AMR surveillance programs in most countries primarily focus on human health or livestock. Companion animals, despite their close proximity to humans, are rarely included. This surveillance gap delays the recognition of emerging threats and hinders proactive intervention. Integrating companion animal data into One Health AMR monitoring frameworks would allow for earlier detection of zoonotic threats and more informed public health strategies [13,59].

Veterinary clinics and hospitals must implement IPC measures analogous to those in human healthcare. This includes barrier nursing, appropriate personal protective equipment (PPE), routine screening of high-risk patients, and evidence-based disinfection protocols to prevent nosocomial outbreaks of MDR pathogens [5,66]. Strengthening IPC practices is critical to halting local transmission within and between species.

Veterinary AMS is a cornerstone of One Health efforts. The empirical use of critically important antimicrobials (CIAs), including carbapenems, colistin, and third-generation cephalosporins, should be minimized. Evidence-based prescribing guided by culture and sensitivity testing, along with veterinary-specific guidelines, are essential tools for slowing resistance development [59,60].

The environment also plays a role in the transmission ecology of *K. pneumoniae*. Shared spaces, such as urban parks, animal boarding facilities, and wastewater systems, may serve as reservoirs and mixing grounds for resistant strains. Multisectoral collaborations between veterinarians, clinicians, microbiologists, and environmental scientists are vital for mapping transmission routes and formulating effective control measures [4,8,20,56,57].

Efforts to combat zoonotic AMR threats must extend to the general public. Awareness campaigns targeting pet owners can encourage responsible antibiotic use, hygiene practices, and timely veterinary consultation. Veterinary professionals should be empowered with resources to educate clients about the broader health implications of resistant infections [67,68,69,70,71].

The zoonotic potential of *K. pneumoniae* is no longer speculative; it is molecularly documented and epidemiologically relevant [63,72,73,74,75,76]. A coordinated, interdisciplinary response under the One Health umbrella is essential to detect, prevent, and control the spread of MDR *K. pneumoniae* at the animal–human-environment interface. This requires closing surveillance gaps, enforcing stewardship, and fostering cross-sectoral collaboration at both local and global levels [63,67,74,75,77].

## 8. Alternative and Adjunctive Strategies

The rise of MDR *K. pneumoniae* in dogs has renewed interest in therapeutic strategies that either reduce reliance on conventional antibiotics or enhance their efficacy. In addition to optimizing antimicrobial stewardship, several alternative or adjunctive approaches are being explored, including bacteriophage therapy, antimicrobial peptides, anti-virulence agents, microbiome-based interventions, and strengthened infection prevention measures. Most data derive from experimental models or human medicine, but they provide a proof-of-concept that is highly relevant to veterinary practice. For companion animals, such strategies may be particularly attractive in situations where conventional options are limited (e.g., ESBL or carbapenemase-producing strains) or where long-term suppression of colonization rather than cure is the goal (e.g., chronic carriers in multi-pet households) [78,79].

### 8.1. Bacteriophage Therapy

Bacteriophages targeting *K. pneumoniae* have shown encouraging results in vitro and in vivo, including lysis of MDR and hypervirulent strains, biofilm disruption, and improved survival in experimental infections. Recent reviews and preclinical studies highlight the capacity of lytic phages and phage cocktails to reduce bacterial burden in pneumonia, sepsis, and wound models, including strains carrying ESBLs or carbapenemases (e.g., KPC, NDM) [80,81]. Combination regimens, in which phages are administered alongside β-lactams or colistin, often show synergistic effects and delayed emergence of resistance, suggesting their potential role as adjuncts rather than stand-alone agents [82].

In veterinary medicine, phage therapy remains largely experimental, but accumulating evidence in livestock and companion animals indicates feasibility and general safety. Reviews of phage use in animals describe successful treatment or prophylaxis of diverse bacterial infections and emphasize advantages such as high specificity, self-amplification at the infection site, and limited impact on the commensal microbiota [83,84]. Reports and opinion surveys suggest that both veterinarians and pet owners may accept phage therapy as an alternative antimicrobial, provided that regulatory frameworks, quality control, and communication about risks and benefits are robustly addressed. Although published clinical data on phage therapy specifically for canine *K. pneumoniae* infections are still lacking, these experiences support further translational research and potential compassionate-use protocols for refractory MDR cases in dogs [84,85].

### 8.2. Antimicrobial Peptides

Antimicrobial peptides (AMPs) are another promising option against MDR *K. pneumoniae*, including strains resistant to last-line drugs such as polymyxins and carbapenems. Several AMPs, such as K11, LI14, Cec4, and other synthetic or engineered peptides, have demonstrated potent bactericidal and antibiofilm activity against clinical *K. pneumoniae* isolates, often with synergy when combined with conventional antibiotics [86,87]. In canine medicine, AMPs are not yet available as licensed systemic therapies, but topical or local applications (e.g., wound or otic preparations) and future systemic formulations could become valuable adjuncts for difficult *K. pneumoniae* infections [88].

### 8.3. Anti-Virulence and Novel Targeted Approaches

Targeting virulence rather than viability, through capsule depolymerases, siderophore or quorum-sensing inhibitors, and other anti-virulence agents, offers a strategy to “disarm” *K. pneumoniae* while exerting less selective pressure for classical resistance. Phage-derived depolymerases and small-molecule inhibitors have been shown to reduce capsule integrity, impair biofilm formation, and enhance susceptibility to host immunity and antibiotics [89,90]. Parallel work on CRISPR-based antimicrobials has demonstrated that endogenous or engineered CRISPR-Cas systems can selectively eliminate resistance plasmids or specific AMR genes in *K. pneumoniae*, effectively re-sensitizing strains to β-lactams in experimental models [91]. While these approaches are far from clinical implementation in dogs, they illustrate future adjunctive strategies that could complement antibiotics and reduce the burden of MDR lineages at the animal–human interface.

Host-directed approaches, such as immunomodulatory agents, vaccines, or monoclonal antibodies, aim to enhance the dog’s ability to control *K. pneumoniae* infection rather than directly targeting the bacterium. Experimental work in humans and animal models is exploring vaccines against capsular or O-antigen components, as well as monoclonal antibodies that neutralize key virulence determinants, with early data indicating protection against invasive disease and reduction in bacterial loads [90]. Although no licensed vaccines or immunotherapies currently exist for canine *K. pneumoniae*, these strategies align with the broader One Health goal of reducing antibiotic use through enhanced host resilience, and they may become relevant in the future for dogs at high risk of recurrent or nosocomial infections.

### 8.4. Microbiome-Based Interventions

Emerging evidence indicates that manipulation of the intestinal microbiota can help eradicate or suppress MDR *K. pneumoniae* carriage, which is particularly relevant given the role of gastrointestinal colonization as a reservoir for extraintestinal infections. Fecal microbiota transplantation (FMT) has been successfully used in human patients to clear ESBL or carbapenem-resistant *K. pneumoniae* and other Enterobacterales from the gut, often with concurrent resolution of recurrent urinary tract infections or pneumonia [92,93]. Systematic reviews and meta-analyses suggest that FMT, probiotics, and selective digestive decontamination can increase decolonization rates of ESBL or carbapenem-resistant Enterobacterales compared with spontaneous loss alone, although optimal protocols and long-term safety remain uncertain [79,94]. In dogs, controlled data are not yet available, but extrapolating from these human studies, microbiome-based interventions may eventually play a role in managing chronic MDR *K. pneumoniae* carriers in high-risk settings such as referral hospitals or immunocompromised patients.

### 8.5. Antimicrobial Stewardship and Infection Prevention

Regardless of the availability of novel agents, rigorous antimicrobial stewardship remains the most immediately impactful “adjunctive” strategy to curb MDR *K. pneumoniae* in dogs. Guidelines from veterinary and international organizations emphasize judicious use of CIAs, culture-based therapy, and practice-level stewardship programs that track and feedback prescribing data [95]. Interventions such as decision-support tools, prescribing audits, and client education have been associated with meaningful reductions in antimicrobial use in small-animal practice without compromising clinical outcomes. For *K. pneumoniae* specifically, stewardship translates into avoiding unnecessary broad-spectrum therapy, stepping down treatment based on susceptibility results, and reserving carbapenems or new β-lactam/β-lactamase inhibitor combinations for truly last-resort situations [96].

Infection prevention and control (IPC) measures in veterinary hospitals complement stewardship by reducing the incidence and spread of *K. pneumoniae* infections and colonization. Evidence from human healthcare and emerging veterinary studies highlights the importance of barrier nursing, hand hygiene, environmental cleaning (including sinks and drains), and appropriate management of indwelling devices in limiting transmission of ESBL and carbapenem-resistant *K. pneumoniae* [97,98]. For dogs, practical IPC measures include standardized catheter-care bundles, isolation of patients colonized or infected with MDR organisms, routine cleaning and disinfection protocols in intensive care and surgical wards, and integration of companion animals into One Health surveillance networks. By reducing infection pressure, these “non-drug” interventions function as critical adjuncts that enhance the effectiveness of both conventional antibiotics and emerging alternative therapies [99,100].

Taken together, alternative and adjunctive strategies against *K. pneumoniae* in dogs span a continuum from near-clinical options (phage therapy in compassionate-use frameworks, improved stewardship and IPC) to more experimental approaches (AMPs, anti-virulence agents, CRISPR-based antimicrobials, microbiome engineering, and vaccines). While robust clinical data in canine populations are still scarce, the convergence of evidence from human medicine, experimental models, and broader veterinary applications highlights their potential to mitigate the impact of MDR and hypervirulent lineages at the animal–human interface [95,96,97,98]. Future priorities include establishing safety and efficacy of phage or AMP-based therapies in dogs, evaluating microbiome-targeted decolonization strategies in high-risk patients, and embedding these innovations within rigorous stewardship and surveillance programs. Such an integrated approach may ultimately allow clinicians to preserve the utility of existing antibiotics while progressively incorporating targeted alternatives into small-animal practice.

While experimental studies and human clinical experience provide encouraging proof-of-concept for alternative and adjunctive strategies against *K. pneumoniae*, their translation into routine veterinary practice remains limited. Most evidence supporting bacteriophage therapy, antimicrobial peptides, anti-virulence approaches, and microbiome-based interventions originates from in vitro models, experimental infections, or human medicine [1,2,10]. In dogs, robust clinical data addressing safety, efficacy, optimal dosing, and long-term outcomes are largely lacking. Additional barriers include regulatory uncertainty, limited availability of standardized products, and the absence of veterinary-specific guidelines for clinical use [14,15]. Consequently, these approaches should currently be regarded as experimental or investigational, highlighting the need for well-designed veterinary clinical trials and regulatory frameworks before widespread implementation in canine medicine [3].

## 9. Prevention and Control Measures

The increasing prevalence of multidrug-resistant (MDR) and potentially zoonotic *K. pneumoniae* strains in dogs highlights the need for effective prevention and control strategies guided by One Health principles, integrating veterinary, human, and environmental health approaches [63,66,67,72,73,101,102,103].

Veterinary hospitals and clinics play a central role in limiting nosocomial transmission. Reports of MDR *K. pneumoniae* in hospitalized animals highlight the importance of robust infection prevention and control measures, including hand hygiene, environmental disinfection, isolation of infected or colonized animals, appropriate sample handling, and waste management [11,60,66]. Ongoing staff training and compliance monitoring are essential to sustain these efforts [66].

Antimicrobial stewardship is critical, as antimicrobial overuse remains a major driver of resistance development [104]. Stewardship programs are vital to preserve the efficacy of existing drugs and reduce the emergence of MDR pathogens [59,60].

Recommended practices include culture and susceptibility testing prior to therapy, avoidance of critically important antimicrobials unless no alternatives exist [13], preference for narrow-spectrum treatments, and education of veterinarians and pet owners. National and international guidelines, including those from the European Medicines Agency, should guide local stewardship policies.

Surveillance remains inconsistent globally, emphasizing the need for integrated frameworks that combine veterinary and human health data. Key components include veterinary AMR databases, whole-genome sequencing to identify high-risk clones, and reporting of unusual or severe cases under One Health initiatives [8,11,45].

Pet owners may also contribute to the dissemination of resistant pathogens. Simple hygiene measures, adherence to prescribed antimicrobial regimens, and targeted awareness campaigns—particularly for multi-pet households and immunocompromised individuals—are important risk-reduction strategies [4,8,56,64].

The prevention of *K. pneumoniae* transmission and resistance development requires a combination of responsible antimicrobial use, hygiene measures, surveillance, and cross-sector collaboration [9,76,105,106,107,108,109,110,111,112]. As companion animals increasingly share our homes and lifestyles, the veterinary community must take a leading role in mitigating zoonotic risks and preserving antibiotic efficacy for both humans and animals.

The escalating global threat posed by multidrug-resistant (MDR) *Klebsiella pneumoniae* has catalyzed renewed interest in bacteriophage (phage) therapy as an alternative or adjunct to conventional antimicrobial treatments. Bacteriophages are viruses that selectively infect and lyse specific bacterial hosts, offering a targeted approach that minimizes disruption to the commensal microbiota. This precision is particularly advantageous in managing *K. pneumoniae* infections, which are often resistant to multiple antibiotic classes and are capable of forming biofilms [113,114].

Recent in vivo and in vitro studies have demonstrated the efficacy of phage cocktails in reducing bacterial burden and inhibiting biofilm-associated MDR *K. pneumoniae* strains [115,116]. Although veterinary applications are still emerging, compassionate-use cases have been reported in dogs with recalcitrant infections. These early experiences highlight the therapeutic potential of phages in veterinary medicine, particularly where traditional antimicrobials have failed.

Nonetheless, several challenges remain. These include the rapid development of phage resistance, the need for highly specific matching between phage and bacterial host, difficulties in regulatory approval, and manufacturing constraints for clinical-grade preparations. Despite these hurdles, the integration of phage therapy into veterinary practice, particularly for zoonotic pathogens such as *K. pneumoniae*, aligns strongly with One Health objectives [113,114]. By diversifying treatment strategies and reducing reliance on conventional antibiotics, phage-based approaches may serve as a critical component in mitigating antimicrobial resistance at the human–animal interface.

## 10. Future Directions and Research Needs

While substantial progress has been made in characterizing *K. pneumoniae* in human healthcare settings, our understanding of its epidemiology, pathogenesis, and zoonotic potential in dogs remains fragmented [117,118,119]. The convergence of antimicrobial resistance and virulence traits in canine isolates poses a complex challenge that urgently requires targeted research and coordinated surveillance [120,121]. Addressing these knowledge gaps will strengthen One Health preparedness and veterinary public health strategies.

Most current data on *K. pneumoniae* in dogs derive from isolated case reports or small-scale studies [4,6,11]. Broader multicenter studies are needed to (i) determine prevalence and colonization rates in healthy versus clinical populations, (ii) identify geographic, seasonal, or demographic patterns in infection and resistance, and (iii) assess the role of environmental and socio-behavioral factors in transmission dynamics.

Expanding the use of WGS and comparative genomics in veterinary isolates will allow researchers to track the spread of international high-risk clones (e.g., ST11, ST15, ST307) in animal populations [5], detect novel resistance determinants and plasmid types, and investigate horizontal gene transfer between commensals and pathogens in dogs, as well as their human contacts [8]. Such a molecular insight will support more targeted infection control and stewardship policies.

### 10.1. Experimental Infection Models

There is a lack of standardized animal models for studying *K. pneumoniae* infection dynamics in dogs. Future work should aim to develop in vivo and ex vivo models to explore host–pathogen interactions, especially for hypervirulent and MDR strains, investigate the immunopathogenesis of canine infections and their potential systemic effects, evaluate the fitness cost and transmissibility of plasmid-encoded resistance and virulence genes in canine hosts [24,44,122].

### 10.2. One Health-Oriented Risk Assessments

Interdisciplinary studies involving microbiologists, veterinarians, clinicians, and epidemiologists are essential to quantify the risk of zoonotic and reverse-zoonotic transmission under real-world conditions (e.g., households, clinics, shelters), map transmission networks using metagenomics and resistome analysis in shared environments, and determine the impact of pet-human interactions on AMR dissemination at the community level [59,60].

### 10.3. Veterinary Antimicrobial Stewardship Frameworks

Guidelines for antimicrobial stewardship in companion animals are still evolving. Future research should evaluate the effectiveness of stewardship interventions on AMR trends in *K. pneumoniae,* develop clinical breakpoints specific to canine pathogens and tissues, and create decision-support tools for veterinarians to guide empiric versus targeted therapy [13].

Comprehensive and collaborative research is essential to clarify the role of dogs in the ecology of *K. pneumoniae*. As our understanding grows, evidence-based policies and interventions will be needed to reduce the pathogen’s impact on animal and human health alike. Investment in One Health-aligned research will not only safeguard antimicrobial efficacy but also reinforce the health of companion animals in modern society [4,8,20,22,56,57].

### 10.4. Limitations of This Review

This is a narrative review and not a systematic synthesis; study selection may therefore be subject to selection and publication bias. Available veterinary data are heterogeneous in design and setting (referral vs. primary care), often with small sample sizes and variable antimicrobial susceptibility testing standards, which limits meta-analytic comparisons. Molecular methods and depth of genomic characterization differ markedly across studies, hindering direct comparison of sequence types, plasmid backbones, and virulence/resistance determinants. Geographical coverage is uneven, with possible under-representation of regions. Finally, evidence for zoonotic and reverse-zoonotic transmission is frequently inferential (e.g., shared STs or plasmids) rather than based on prospective, household-level genomic linkage, so directionality cannot be assumed.

## 11. Conclusions

*Klebsiella pneumoniae* is increasingly recognized as a relevant pathogen in canine medicine, driven by rising antimicrobial resistance and the emergence of hypervirulent lineages. Although disease prevalence in dogs remains relatively low, affected animals may carry strains that mirror those circulating in human populations, including high-risk sequence types and plasmid-borne resistance determinants. This overlap highlights the potential for bidirectional transmission and the importance of a One Health perspective.

Current evidence shows that dogs can act as reservoirs or amplifiers of MDR *K. pneumoniae*, especially in clinical environments or following antimicrobial exposure. Effective management therefore relies on routine susceptibility testing, strengthened infection-control practices, and responsible antimicrobial stewardship. Expanding genomic surveillance and integrating companion animals into AMR monitoring frameworks are essential next steps.

As knowledge gaps persist regarding transmission pathways, colonization dynamics, and host–pathogen interactions, coordinated research efforts are needed to refine risk assessments and guide preventive strategies. Recognizing the interconnected roles of humans, animals, and the environment will be crucial for limiting the spread of *K. pneumoniae* and safeguarding antibiotic efficacy across the veterinary and public health sectors.

## Figures and Tables

**Table 1:** Resistance and virulence summary in *K. pneumoniae* from dogs.

Category	Type/Factor	Associated Genes	Clinical Relevance	Key References
Antimicrobial Resistance	β-lactams (Penicillins, Cephalosporins)	*bla*_SHV, *bla*_TEM, *bla*_CTX-M	Common; causes ESBL production and therapeutic failure	Marques et al. [4,6]; Lee et al. [30]; Frenzer et al. [11]
Antimicrobial Resistance	Carbapenems	*bla*_KPC, *bla*_NDM, *bla*_OXA-48	Rare but critical; resistance to last-resort drugs	Pachanon et al. [5]; Zhang et al. [10];
Antimicrobial Resistance	Aminoglycosides	*aac*(6′)-Ib, *aad*A, *arm*A	Common; limits treatment options for severe infections	Frenzer et al. [11]; Marques et al. [4,6]; Chen et al. [8]
Antimicrobial Resistance	Fluoroquinolones	*qnr*A, *qnr*B, *qnr*S, *gyr*A mutations	Increasing; complicates treatment of respiratory and urinary infections	Pachanon et al. [5]; Chen et al. [8]
Antimicrobial Resistance	Tetracyclines	*tet*(A), *tet*(B)	Frequent in veterinary medicine; resistance compromises utility	Frenzer et al. [11]; Marques et al. [4,6]
Antimicrobial Resistance	Sulfonamides	*sul*1, *sul*2	Observed in polymicrobial infections; adds resistance pressure	Lee et al. [30]; Chen et al. [8]
Virulence Factor	Capsule (K1, K2)	*wzi*, *mag*A	Enhances immune evasion; linked to severe infections	Lam et al. [25]; Pachanon et al. [5]
Virulence Factor	Hypermucoviscosity (HMV)	*rmp*A, *rmp*A2	Linked with invasiveness and metastatic infections	Pachanon et al. [5];
Virulence Factor	Fimbriae and Adhesins	*fim*H, *mrk*D	Promotes colonization and persistence, especially in UTIs	Struve et al. [26]; Chen et al. [8]
Virulence Factor	Siderophores	*ent*B, *iut*A, *iro*N, *ybt*S	Critical for survival in host; associated with hypervirulence	Holden et al. [27]; Pachanon et al. [5]
Virulence Factor	Lipopolysaccharide (LPS)	LPS biosynthesis cluster (*waa*, *rfa* genes)	Triggers inflammation and contributes to septic shock	Santajit and Indrawattana [2]

## Data Availability

No new data were created or analyzed in this study. Data sharing is not applicable to this article.
